# Genotype-phenotype association analysis identifies the role of α globin genes in modulating disease severity of β thalassaemia intermedia in Sri Lanka

**DOI:** 10.1038/s41598-019-46674-y

**Published:** 2019-07-12

**Authors:** Shiromi Perera, Angela Allen, Ishari Silva, Menaka Hapugoda, M. Nirmali Wickramarathne, Indira Wijesiriwardena, Stephen Allen, David Rees, Dimitar G. Efremov, Christopher A. Fisher, David J. Weatherall, Anuja Premawardhena

**Affiliations:** 1 Faculty of Medicine, University of Kelaniya, Kelaniya, Sri Lanka; 2 MRC Molecular Hematology Unit, Weatherall Institute of Molecular Medicine, University of Oxford, Oxford, UK; 3 Hemal’s Thalassemia Care Unit, North Colombo Teaching Hospital, Ragama, Sri Lanka; 4 Faculty of Applied Sciences, Sabaragamuwa University of Sri Lanka, Belihulova, Sri Lanka; 5 Faculty of Medicine, University of Sri Jayewardhanepura, Nugegoda, Sri Lanka; 6 Centre for Tropical and Infectious Disease, Liverpool School of Tropical Medicine, Liverpool, UK; 7 Department of Paediatric Haematology, King’s College Hospital, London, UK; 8 Molecular Hematology Unit, International Centre for Genetic Engineering and Biotechnology, Rome, Italy

**Keywords:** Molecular medicine, Genetics research

## Abstract

β thalassaemia intermedia (βTI) are a heterogeneous group of disorders known to be extremely phenotypically diverse. This group is more complex to manage as no definitive treatment guidelines exist unlike for β thalassaemia major (βTM). There are only a few studies looking at genotype phenotype associations of βTI outside the Mediterranean region. The reasons for the diverse clinical phenotype in βTI are unknown. We categorized fifty Sri Lankan patients diagnosed with βTI as mild, moderate or severe according to published criteria. DNA samples were genotyped for β thalassaemia mutations, α globin genotype and copy number and known genetic modifiers of haemoglobin F production. There were 26/50 (52.0%) in mild group and 12/50 (24.0%) each in moderate and sever categories. 18/26 (69.2%) classified as mild were β heterozygotes and 17/18 (94.4%) had excess α globin genes. 11/12 (91.6%) classified as moderate were β heterozygotes and 8/11 (72.2%) had excess α globin genes. In contrast, 8/12 (66.7%) classified as severe were β homozygotes and 7/8(87.5%) had α globin gene deletions. In Sri Lanka, co-inheritance of either excess α globin genes in β thalassaemia heterozygotes or α globin gene deletions in β thalassaemia homozygotes is a significant factor in modulating disease severity.

## Introduction

The haemoglobin (Hb) disorders, including thalassaemia and sickle cell disease are the most commonly inherited blood diseases in the world and cause a major public health problem, particularly in resource poor countries across the tropical belt^1^. Beta (β) thalassaemia is heterogeneous, both in terms of its genetic basis and clinical phenotype. Individuals who inherit a single mutated β globin allele are classified as “Thalassaemia Trait” (TT) and are usually clinically asymptomatic, although they may have mild anaemia. Individuals who inherit two mutant β globin alleles are classified as “Thalassaemia Major” (TM) and most are blood transfusion dependent^2^. The term β thalassaemia intermedia (βTI) describes patients in whom disease severity lies between TT and TM. These patients are defined clinically with no universally accepted criteria.

The underlying pathophysiology of β thalassaemia is related to a quantifiable deficiency of functional β globin chains, which leads to an imbalanced globin chain production. In β thalassaemia the synthesis of normal α globin chains from the unaffected globin genes continues as normal, resulting in the accumulation of excess unmatched α globin chains within the erythroid precursors. The excess α globin chains are unable to form viable tetramers which instead precipitate in the red cell precursors in the bone marrow forming inclusion bodies. This leads to premature intramedullary destruction of erythroid precursors and causes ineffective erythropoiesis in β thalassaemia^2^. Apart from the degree of β globin chain deficiency, the globin chain imbalance can also be influenced by the level of α and gamma (γ) globin chain production. Co-inheritance of α thalassaemia (which reduces the excess α globin chain) and/or genetic factors which increase the γ globin gene output can ameliorate the clinical course of the disease^2–6^.

There are several genetic factors which can influence the phenotype of β thalassaemia. The “primary modifier” of disease severity is the varying expression of β thalassemia alleles. Co inheritance of variations of alpha (α) globin gene copy number as well as modifiers which augment fetal hemoglobin (Hb F) production are referred to as secondary modifiers^7–10^. Factors which directly have no bearing on the globin chain balance but nevertheless influence the clinical phenotype are referred to as the tertiary modifiers. The final phenotype of a patient is affected by environmental and psychosocial factors too^1–3^.

In Sri Lanka, the majority of patients with haemoglobinopathies seeking treatment have either βTM or haemoglobin E β thalassaemia^11–15^. We describe the clinical phenotype and genetic basis of βTI in a case series of Sri Lankan patients and assess genetic factors associated with disease severity.

## Results

**Figure 1:**
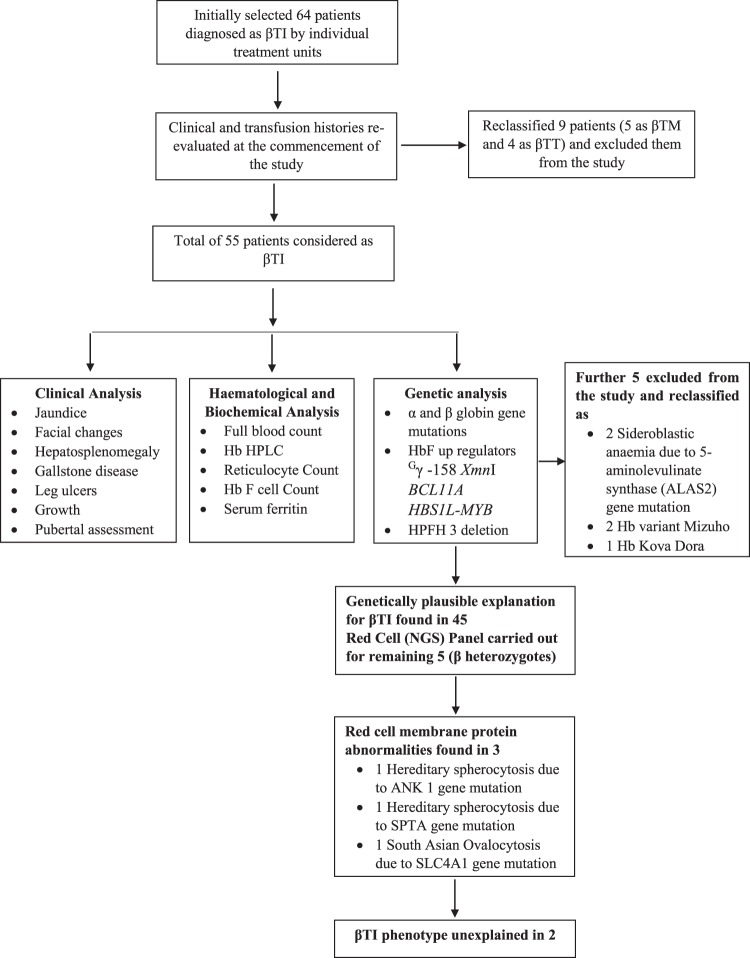
Graphical presentation of the work flow.

The graphical abstract of the work flow is summarized in Fig. 1. A total of 64 patients classified as βTI by his or her treating haematologist or clinician were recruited. Based on clinical features, baseline haemoglobin concentrations and transfusion requirements, 9 patients were re-classified; four as β TT and five who had required blood transfusion more frequently than every 6 weeks as βTM. Following DNA analyses a further 5 patients without β–thalassaemia mutations were also reclassified: two as sideroblastic anaemia, 2 as unstable haemoglobin variants Hb Mizuho and 1 case of Hb Koya Dora. These 14 patients were excluded from the analysis.

Of the remaining 50 patients, 35 (70.0%) were recruited from Ragama thalassaemia centre, 5 (10.0%) Chilaw, 4 (8.0%) Kurunegala, 4 (8.0%) Badulla and 2 (4.0%) from Anuradhapura thalassaemia centres. Age ranged from 3–65 years (median 23.5; IQR = 12.8–38.2 years) and 32/50 (64.0%) were female. Forty-nine patients (98.0%) were of Sinhalese ethnicity and one was Muslim. In three families there was more than one affected family member: two siblings in one family and a parent and a child in two families.

### Clinical findings

Demographic and clinical findings according to disease severity are summarized in Table 1. In accordance with previously published guidelines^16^ 26 (52%) individuals were classified as phenotypically mild, 12 (24%) as moderate and 12 (24%) as severe. In accordance with the criteria used to classify disease severity, younger age at first presentation and first transfusion and greater frequency of blood transfusions were associated with severe disease. Clinical features associated with more severe disease were splenomegaly, hepatomegaly, severe facial changes and requirement for iron chelation therapy. None of the patients had any events suggestive of superficial thrombophlebitis, deep vein thrombosis, portal venous thrombosis or pulmonary embolism. None had a history of fractures, had suffered an overt stroke or episodes suggestive of peripheral vascular disease or acute limb ischemia.

**Table 1:** Clinical Findings of β thalassemia intermedia cohort in Sri Lanka.

Clinical Findings	All β thalassemia intermedia n = 50	β thalassemia intermedia disease severity			P value
		Mild n = 26	Moderate n = 12	Severe n = 12	
Age at first presentation (years)	8.5 (5.0–26.0)	10.0 (5.4–25.8)	19.5 (6.0–46.2)	4.0 (0.83–8.0)	0.010
No. patients received > = 1 blood transfusion	43/50 (86%)	19/26 (38%)	12//12 (100%)	12/12 (100%)	0.021
Age at first transfusion (years)	10.5 (5.0–28.8)	17.0 (10.0–31.0)	19.5 (6.5–51.2)	4.0 (2.3–8.0)	0.007
Average No. blood transfusions per year	1.3 (0.31–5.0)	0.41 (0.0–1.2)	3.3 (1.2–4.6)	7.8 (5.9–9.0)	<0.001
Age at clinical review (years)	23.5 (12.8–38.2)	25.5 (15.2–38.5)	37.5 (18.8–57.8)	18.0 (6.8–22.8)	0.014
Splenectomised	6/50 (12.0%)	2/26 (7.7%)	2/12 (16.7%)	2/12 (16.7%)	0.605
Splenomegaly	30/44 (68.2%)	11/24 (45.8%)	10/10 (100.0%)	9/10 (90.0%)	0.002
Spleen size (cm)	7.0 (4.8–10.2)	6.0 (9.0–13.0)	4.5 (2.8–8.0)	8.0 (5.0–10.0)	0.110
Hepatomegaly	19/50 (38.0%)	6/26 (23.1%)	4/12 (33.3%)	9/12 (75%)	0.010
Jaundice	34/50 (68.0%)	21/26 (80.8%)	10/12 (83.3%)	3/12 (25.0%)	0.002
Gall stones	5/50 (10.0%)	3/26 (11.5%)	2/12 (16.7%)	0/12 (0.0%)	0.410
Cholecystectomy	3/50 (6.0%)	2/26 (7.7%)	1/12 (8.3%)	0/12 (0.0%)	0801
Leg ulcers	4/50 (8.0%)	2/26 (7.7%)	2/12 (16.7%)	0/12 (0.0%)	0.526
Severe Facial changes	7/50 (14%)	2/26 (7.7%)	0/12 (0.0%)	5/12 (41.7%)	0.011
Pulmonary Hypertension					
No pulmonary Hypertension	26/39 (66.7%)	9/19 (34.6%)	6/9 (50%)	11/11 (91.7%)	0.004
Mild pulmonary Hypertension (PAP 25–40 mmHg)	13/39 (33.3%)	10/19 (38.5%)	3/9 (25%)	0/11 (0%)	0.029
BMI	17.6 (15.3–21.0)	18.7 (15.8–20.7)	17.2 (14.7–19.9)	16.6 (14.4–19.2)	0.112
Completed Growth	35/50 (79%)	19/26 (73.1%)	9/12 (75.0%)	7/12 (58.3%)	0.71
Age appropriate Tanner staging	37/50 (74.0%)	22/26 (84.6%)	9/12 (75.0%)	6/12 (50.0%)	0.091
Delayed puberty	6/50 (12.0%)	1/26 (3.8%)	2/12 (16.7%)	3/12 (25.0%)	0.113
Children with Tanner 1 (3–8 years)	7/50 (14%)	3/26 (11.5%)	1/12 (8.3%)	3/12 (25%)	0.486
Number on Iron chelation	23/50 (46%)	6/26 (23%)	6/12 (50%)	11/12 (91.6%)	<0.001

Data are median (IQR) or number (%).

Thirty-five patients had completed growth. The final height was below the 3^rd^ centile in 2/35 (5.7%) and between the 3^rd^ and the 25^th^ centile in a further 3/35 (8.6%) whilst it had surpassed the 50^th^ centile in 30/35 (85.7%). In the remaining 15 who were still growing the values were below the 3^rd^ centile in 4/15 (26.7%), between the 3^rd^ and the 25^th^ centile in 6/15 (40.0%), between 25^th^ and the 50^th^ centile in 2/15 (13.3%) and over the 50^th^ centile in 3/15 (20%).

Thirty-seven patients (37/50, 74%) had age appropriate Tanner staging. Puberty was delayed in 6/50 (12%), two of whom had height and weight below 3^rd^ centile. Two patients were receiving hormone replacement therapy and puberty was induced in one other. Median age at menarche was 14 years (n = 16; IOR = 12–14 years). Eleven females and nine males had married and had offspring.

### Transfusion history

On review of the transfusion history, 7/50(14%) had never been transfused, 21/50(42%) had one transfusion per year or less, 12/50(24%) had two to five transfusions per year and 10/50(20%) had ≥ 6 transfusions per year and had varying off transfusion periods (3 months to 4 years). The median age at first transfusion was 10.5 years (IQR = 5.0–28.8 years) and the median Hb at the first transfusion was 7.0 g/dl (IQR = 6.0–7.5 g/dl).

The laboratory findings are summarized in Table 2.

**Table 2:** Laboratory Findings of β thalassemia intermedia cohort in Sri Lanka.

Laboratory Findings	All β thalassemia intermedia n = 50 Median (IQR)	β thalassemia intermedia disease severity			P value
		Mild n = 26 Median (IQR)	Moderate n = 12 Median (IQR)	Severe n = 12 Median (IQR)	
Hb at first presentation (g/dl)	(n = 45) 7.3 (6.2–8.5)	(n = 26) 7.6 (6.8–8.7)	(n = 10) 6.8 (6.0–8.2)	(n = 9) 7.0 (5.6–8.1)	0.725
Steady state Hb (g/dl)	(n = 49) 7.9 (6.9–8.3)	(n = 25) 8.1 (7.8–9.0)	(n = 12) 7.1 (6.6–7.8)	(n = 12) 7.7 (6.8–8.0)	0.001
MCV (fl)	(n = 50) 67.8 (62.6–74.9)	(n = 26) 64.5 (60.2–70.0)	(n = 12) 75.0 (66.5–81.3)	(n = 12) 68.4 (67.3–76.4)	0.077
MCH (pg)	(n = 50) 21.8 (20.3–25.1)	(n = 26) 20.8 (19.8–23.0)	(n = 12) 25.4 (22.0–27.3)	(n = 12) 21.2 (20.0–25.2)	0.029
HPLC: HbA2 (%)	(n = 48) 4.6 (3.6–5.2)	(n = 25) 4.7 (3.3–5.6)	(n = 12) 4.6 (3.7–5.2)	(n = 11) 4.5 (2.8–5.0)	0.555
HPLC: HbF (%)	(n = 49) 5.8 (1.8–16.3)	(n = 26) 4.6 (1.6–18.6)	(n = 12) 2.9 (1.4–10.0)	(n = 11) 10.1 (6.7–94.6)	0.041
Reticulocyte count (%)	(n = 45) 2.0 (1.6–3.1)	(n = 25) 2.0 (1.8–2.8)	(n = 10) 3.1 (1.6–3.6)	(n = 10) 1.8 (1.0–3.2)	0.376
HbF cell count (%)	(n = 46) 2.6 (1.2–9.3)	(n = 25) 2.0 (1.2–12.2)	(n = 12) 2.6 (1.2–5.4)	(n = 9) 6.0 (1.2–90.0)	0.507
Ferritin (ng/ml)	(n = 48) 294.8 (147.2–503.6)	(n = 25) 168.0 (62.3–294.3)	(n = 12) 406.9 (301.4–821.3)	(n = 11) 477.1 (292.0–670.6)	0.001

Data are median (IQR) or number (%).

All patients were anaemic when they first presented: median Hb of 7.9 g/dl (IQR = 6.9–8.3 g/dl). Steady state Hb was lowest, and red cell indices greatest in the moderate group (median MCV 75.0 fl (IQR = 66.5–81.3 fl) and median MCH 25.4 pg (IQR = 22.0–27.3 pg). HbF levels and HbF cell counts were greatest in the severe group, as was serum ferritin concentration.

### Genetic findings

In total, 12 β globin mutations were identified, 9 of which were β^0^ and β^+^ alleles, The IVSI-5 (G → C) and IVSI-1 (G → A) were the most common. A transcriptional mutation −90 (C → T) in the promoter region of the β globin gene was identified in one patient, two patients inherited the rare haemoglobin variants Hb G-Szuhu (HBB: c.243 C > G) and Hb G-Coushatta (HBB: c.68 A > C), and HbF up-regulators *Xmn*I ^G^γ+/+ and BCL*11A*+/+ were present in 5/50 (10%) and 1/50 (2%), respectively.

The genotypic factors according to disease severity are summarized in Tables 3 and 4. Of the 26 patients in the mild group, 8 were homozygotes (two mutated β alleles). Of these, 5 had mutant β globin alleles known to be mild and 1 presented with a single α globin gene deletion. The remaining two homozygotes were siblings who both had hereditary persistence of fetal haemoglobin type 3 deletion. Amongst the 18 β heterozygotes with mild disease, (17; 94.4%) had five to six copies of the α globin gene. The remaining heterozygote had a normal α globin gene copy number and had co-inherited a mutation in ANK1 gene associated with Hereditary Spherocytosis (HS).

**Table 3:** Genetic Findings of β thalassemia intermedia cohort in Sri Lanka

Genetic findings	β thalassemia intermedia disease severity		
	Mild	Moderate	Severe
**β thalassemia heterozygotes (n = 33)**			
β thalassemia heterozygote and excess α globin chains β thalassemia heterozygote and with membranopathies β thalassemia heterozygote (Unexplained)*	17 1	8 2 1	3 1
Total	18	11	4
**β thalassemia homozygotes (n = 17)**			
β thalassemia homozygote and α globin gene deletions β thalassemia homozygote with mild β alleles β thalassemia homozygote with Hb F modifiers β thalassemia homozygote HPFH type 3 deletion	1 5 2	1	7 1
Total	8	1	8

^*^Unable to explain genetic mechanism for the phenotypic diversity from the available genetic data.

**Table 4:** Genotypes of 50 Sri Lankan βTI patients, and their association with disease severity.

α globin genotype	β globin genotype	Xmn I ^G^γ	Disease severity		
			Mild (n = 26)	Moderate (n = 12)	Severe (n = 12)
αα/αααα	IVSI −130 G → C		2		
	CD 15 –T		1	1	
	CD 16 - C			2	
ααα/ααα	CD 15 G → A		1		
	IVSI-1 G → A			1	1
αα/ααα	IVSI-1 G → A		8		1
	CD 15 G → A		1		
	CD 16 - C		1		
	IVSI-5 G → C		3	2	1
	CD41/42 – CTTT			2	
αα/αα	IVSI- 5 G → C/Hb G Coushatta	+/+	1		
	CD 30 G → C/CD 30 G → C	+/+	1		
	−90 C → T/−90 C → T^a^	+/+	1		
	IVSI- 5 G → C/Hb G Szuhu		1		
	CD 30 G → C/CD 30 G → C		1		
	IVSI-5 G → C/HPFH3		2		
	IVSI-5 G → C		1	2	1
	CD 15 -T			1	
	IVSI-5 G → C/IVSI-5 G → C	+/+			1
−α^3.7^/αα	IVSI-5 G → C/IVSI-5 G → C		1		2
	CD 15 –T/IVSI −1 G → A			1	
	IVSII-1 G → A/IVSII-1 G → A	+/+			1
	IVSI- I G → A/−90 C → T				1
−α^4.2^/αα	CD 16 - C/CD 16 - C				1
−α^3.7^/−α^3.7^	CD41/42–TTCT/CD41/42 TTCT				1
−α^3.7^/−α^4.2^	IVSI-5 G → C/IVSI-5 G → C				1

Notes ^a^patient also had BCL11A.

Of the 12 patients in the moderate group, 11 were β heterozygotes, 8 (73%) of whom had excess α globin genes. Of the 3 heterozygotes with a normal copy number of α globin genes one had co-inherited a mutation in the SPTA1 gene associated with HS, and another had co-inherited the SLC4A1 variant for south Asian Ovalocytosis. One patient was a β compound heterozygote with a single α globin gene deletion.

Of the 12 patients in the severe group, 8 (66.7%) were β homozygotes/compound heterozygotes, 7 of whom had one or two α globin gene deletions and 1 patient had the ^G^γ - 158 *Xmn*I+/+ polymorphism. There were 4 (33.3%) β heterozygotes, three of whom also had excess α globin genes.

## Discussion

Our study highlights the difficulties in the classification of βTI. Although the patients recruited to our study were managed and classified by clinicians in specialist centres, the diagnosis was subsequently changed in 14, as a result of the clinical assessment of 9 patients and genetic analyses in 5.

Variability in transfusion regimens between the different clinical groups most likely accounted for the fluctuations in the clinical and haematological data between the different severity groups.

Based on the pathophysiology of β thalassaemia, variability in clinical severity can be explained by several genetic mechanisms: inheritance of mild β thalassaemia alleles, co-inheritance of α thalassaemia or extra α globin genes and inheritance of genetic determinants for enhanced production of γ globin chains. Inheritance of excess α globin genes in β thalassaemia heterozygotes (TT), who are normally clinically silent, resulted in more severe anaemia and even requirement for blood transfusion, such that they fell into the βTI category, but remained at the milder end of the βTI disease spectrum. Conversely co- inheritance of α thalassaemia in β thalassaemia homozygotes reduced disease severity, but they tended to lie at the more severe end of the βTI disease spectrum. The results of our analysis are discussed in the context of these ameliorating or exacerbating factors.

Sri Lankan βTI patients were clinically milder, less frequently transfused, had fewer splenectomies and a lesser degree of iron over- load than that described in βTI patients in the Middle East and Mediterranean region. In the Mediterranean region most βTI patients experience more complications; splenectomy 325/584 (55.7%), cholelithiasis 100/584 (17.1%), thrombosis 82/584 (14%), PHT 64/584 (11%), leg ulcers 46/584 (7.9%)^17^. This may be due to differences in classification as discussed previously.

β thalassaemia heterozygotes co-inheriting excess α globin genes accounted for just over half of the cases in our series and most had mild to moderate disease severity. Although co-inheritance of a single β globin gene mutation and excess α globin genes has been reported previously as a genetic basis of βTI in studies from Europe, the Middle East and^4,8,9,18,19^, its frequency was less than in our study, occurring in 10/165 (6.1%) Italian, 3/60 (5%) Israeli and 1/51(2%) Iraqi βTI patients, respectively^9,10,19^. Similar findings have also been reported in studies of β heterozygotes from Asia and South Asia^3,8^: In India 14/23 (60.9%) and in China 5/20 (25%) β heterozygotes co-inherited excess α globin genes, compared to 28/33 (84.8%) in our study.

βTI patients in the Mediterranean comprised mainly of β homozygotes^17^. In India and China, the main genetic causes were co-inheritance of alpha thalassaemia and the presence of the ^G^γ -158 *Xmn*I+/+ polymorphism in β homozygotes, and inheritance of two mild β alleles, respectively^5,7^. Co-inheritance of one or two α globin gene deletions in β homozygotes and compound heterozygotes was the second most common genetic basis of βTI in our patient group, occurring in 9/50 (18%) patients, 7 of whom were in the severe group. Similarly, co-inheritance of α thalassaemia was recognised to be a prominent genetic basis of βTI in India, Italy and Pakistan, occurring in 16/73 (21.9%), 10/74 (19.5%) and 13/63 (20.6%) βTI patients, respectively^5,9,20^. Furthermore, in studies of thalassaemia patients in Cyprus and Sardinia co-inheritance of one or two α globin gene deletions in β homozygotes was the most significant ameliorating factor of disease^6,21^.

Since most β globin gene mutations in β thalassaemia major and haemoglobin E β thalassaemia in Sri Lanka are categorised as severe^11–13^, it is not surprising that in our cohort inheritance of two mild β alleles in βTI was uncommon and accounted for only 5/50 (10%) of cases. This contrasts with studies from Asia and the Mediterranean region, where the frequencies of mild β alleles are much higher; China 41.9%, Lebanon 68.4%, Italy 61.2% and Iraq, 54.9%^7–9,19^.

We identified a transcriptional mutation −90 (C → T) in the promoter region in one patient with a mild phenotype. This is the first time that this mutation has been described in Sri Lanka.

We also identified two patients with rare haemoglobin variants Hb G-Szuhu (HBB: c.243C4G) and Hb G-Coushatta (HBB: c.68A4C) in combination with the common β-thalassaemia mutation, IVS-I-5 (G → C), giving rise to a mild phenotype^22^. Both probands had mild anaemia, greatly reduced red cell indices and splenomegaly that was only identified by ultrasound.

The prevalence of Hb F up-regulators in our group of βTI patients (6/50; 12%) was less than that reported in studies of βTI from other regions in the world. For example, 20/73 (27.3%) Indian and 22/47 (46.8%) Iranian βTI patients were homozygous for the ^G^γ - 158 *Xmn*I polymorphism^5,18^.

Three patients in our study group co-inherited membranopathies, that went undetected by routine examination of thin blood films and were only identified by subsequent advanced genetic analyses. This highlights the intrinsic difficulty in categorizing individuals with βTI, since routine testing may not always identify co existing haematological anomalies and advanced genetic testing facilities may not be available in many resource limited settings.

We were unable to explain the disease severity in two β heterozygotes classified as moderate and severe phenotype suggesting that as yet unidentified genetic determinants and environmental factors may be involved.

### Strengths and weaknesses of our study

There was no selection bias in our patient-based study that recruited all patients previously considered to have βTI from the five main thalassaemia centres in Sri Lanka. During our clinical assessment process, the initial diagnosis was re-evaluated and corrected accordingly. This led to the exclusion of fourteen patients who had been incorrectly classified initially. However, it is also possible that some “true” βTI patients may have been misdiagnosed as βTM at initial diagnosis and may not have been included in our study.

Limited availability of resources and funding meant that the study was confined to an analysis of primary and secondary modifiers of disease severity and we cannot exclude the possibility that other environmental and genetic factors may also have contributed.

In conclusion, we report that the milder clinical phenotype of βTI patients in Sri Lanka compared with those from the Mediterranean/Middle East is due in part to differences in the predominant genetic basis of βTI in each setting. In Sri Lanka, the genetic basis of more than half of βTI patients was co-inheritance of single β globin gene mutations and excess α globin genes. These patients were at the milder end of the βTI disease spectrum.

## Patients and Methods

Between November 2011 and December 2012, study staff visited the five major thalassaemia centres in Sri Lanka; Ragama, Kurunegala, Anuradhapura, Chilaw and Badulla. The clinic notes of 64 patients previously classified as βTI by specialist haematologists/clinicians were reviewed and all were invited to participate in the study. Informed consent was obtained from each patient or the parent/carer if the patients were younger than 18 years.

### Clinical methods

Information regarding family history of thalassaemia, the patient’s age and Hb concentration at the time of diagnosis, frequency of subsequent blood transfusions, gall bladder disease, fractures, recurrent leg ulceration, and hospital admissions with severe infections and thrombotic events was recorded from each patient’s notes. In patients who had received blood transfusion, average steady state Hb was calculated from pre-transfusion Hb values.

The same specialist clinician (IS) examined all patients for physical signs including pallor, jaundice, thalassaemic facial changes and leg ulcers. Each patient’s height, weight and size of the spleen and the liver below the costal margin were measured. Tanner score was used to assess sexual maturity. Age at menarche in females and pubertal induction with hormone preparations were recorded. Details regarding iron chelation and hydroxyurea therapy were also recorded.

Clinical phenotype was categorized as mild, moderate or severe based on transfusion requirements and age when transfusions began, in accordance with published guidelines^16^. Patients who maintained their Hb at 7·5 g/dl or above without transfusion, or with a transfusion frequency of less than once every 2 years, or less than 6 monthly if transfusion was started after age 10 years, were considered as mild. Those patients whose transfusion requirements began at age 4 years or older, with a frequency of between 6 weeks and 4 months, and those who commenced transfusions before the age of 4 years and receiving transfusions every 3 to 4 months were categorized as severe. Those with transfusion requirements between the two groups were classified as moderately affected.

### Laboratory methods

Five ml venous blood was collected from each patient and 2.5 ml was transferred into an EDTA anti-coagulated tube and the remaining sample into a plain tube. The EDTA sample was used for the measurement of haematological parameters (COULTER^®^A^C•^T™ 5 diff., California, USA) and Hb variant analysis by HPLC (Bio-Rad Variant II Analyser, Hercules, CA, USA). Thin blood films were prepared, stained with New Methylene Blue and examined by light microscopy for the presence of reticulocytes. The percentage of HbF cells was determined by the Kleihauer acid elution test^23^.

The remaining sample in the EDTA tube was centrifuged, the buffy coat removed, DNA extracted using standard methods^24^ and stored at −20 °C for genetic analysis.

The sample collected into the plain tube was allowed to clot, centrifuged and the serum removed and used for the measurement of serum ferritin by Enzyme Linked Immunosorbant Assay (ELISA); Kit 16017, Diagnostic Automation Inc, Calabass, Canada).

### Genetic analysis

DNA samples were analysed for the presence of β globin gene mutations by polymerase chain reaction (PCR) and sequencing^25^, and for deletions in the β globin gene cluster that cause hereditary persistence of foetal haemoglobin (HPFH 3), by Gap-polymerase chain reaction (Gap PCR)^26^.

Deletional and non-deletional forms of α thalassaemia were identified by restriction digest and Southern blotting and PCR and sequencing, respectively^27^. Alpha globin copy number was determined by Multiple Ligation Polymerase Amplification (MLPA); Kit P140, MRC-Holland, Amsterdam, Netherlands.

DNA samples were analysed for known genetic modifiers of Hb F production. The presence of the 158 C > T polymorphism in the *Xmn*1 locus of the promoter gene γ 2 by PCR and restriction digest^21^ and single nucleotide polymorphisms (SNPs) in the intergenic regions *HBS1L-MYB* of chromosome 6q23.3 and the *BCL11A* gene on chromosome 2p16, by PCR and sequencing^28^.

DNA Samples were analysed for membranopathies using a next generation sequencing panel, based on the llumina MiSeq system and data was analysed to detect genetic and copy number variants using a bespoke bioinformatics pipeline, validated in the UK. The panel included 147 genes arranged into sub panels for haemolysis, anaemia, and bone marrow failure. Variants were annotated with information from ClinVar, and population frequency data from ExAC and the 1000 genomes project.

Sequence data was analysed using the National Centre for Biotechnology Information (NCBI), Bethesda, MD, USA sequencing analysis software and mutations were identified by comparing sequence data obtained to the globin gene server database; (http://globin.bx.psu.edu./hbvar).

### Statistical analysis

Categorical variables were expressed as counts and percentages and compared using the chi-square test. Continuous variables were expressed as median and inter quartile range and compared using either the Mann-Whitney U test or Kruskall Wallace tests. A p-value < 0.05 was considered statistically significant. All data analysis was performed using Statistical Package for Social Sciences Software (SPSS), version 16.

### Ethical approval

The study was conducted in accordance with the Declaration of Helsinki. The study and the consent procedures were approved by the Ethical Review Committee, University of Kelaniya, Sri Lanka (P95/09/2010) and the Oxford Tropical Research Ethics Committee (OXTREC 515-13).

## Data Availability

All data generated and analysed during this study are included in this published article.
